# Impact of prior and concurrent medication on exacerbation risk with long-acting bronchodilators in chronic obstructive pulmonary disease: a post hoc analysis

**DOI:** 10.1186/s12931-019-1027-9

**Published:** 2019-03-26

**Authors:** Ian Naya, Lee Tombs, David A. Lipson, Isabelle Boucot, Chris Compton

**Affiliations:** 1 Global Respiratory Franchise, GSK, 980 Great West Road, Brentford, Middlesex, TW8 9GS UK; 2 Precise Approach Ltd, contingent worker on assignment at GSK, Stockley Park West, Uxbridge, Middlesex, UK; 3 Respiratory Research and Development, GSK, Collegeville, PA USA; 4 Perelman School of Medicine, University of Pennsylvania, Philadelphia, PA USA

**Keywords:** COPD, Maintenance therapy, Exacerbation risk, ICS, Long acting bronchodilators, Lung function

## Abstract

**Background:**

Symptomatic patients with chronic obstructive pulmonary disease (COPD) and low exacerbation risk still have disease instability, which can be improved with better bronchodilation. We evaluated two long-acting bronchodilators individually and in combination on reducing exacerbation risk and the potential impact of concurrent medication in these patients.

**Methods:**

Integrated post hoc intent-to-treat (ITT) analysis of data from two large 24-week, randomized placebo (PBO)-controlled trials (NCT01313637, NCT01313650). Symptomatic patients with moderate-to-very-severe COPD with/without an exacerbation history were randomized (2:3:3:3) to once-daily: PBO, umeclidinium/vilanterol (UMEC/VI 62.5/25 μg [NCT01313650] or 125/25 μg [NCT01313637]), UMEC (62.5 [NCT01313650] or 125 μg [NCT01313637]) or VI (25 μg) via the ELLIPTA inhaler. Medication subgroups were segmented by treatment status at screening: a) maintenance-naïve or on maintenance medications, b) inhaled corticosteroid [ICS]-free or ICS-treated, c) low or high albuterol use based on median run-in use (< 3.6 or ≥ 3.6 puffs/day). Time to first moderate/severe exacerbation (Cox proportional hazard model) and change from baseline in trough forced expiratory volume in 1 s (FEV_1_; mixed model repeated measures) were analyzed. Safety was also assessed.

**Results:**

Of 3021 patients (ITT population; UMEC/VI: *n* = 816; UMEC: *n* = 825; VI: *n* = 825; PBO: *n* = 555), 36% had a recent exacerbation history, 33% were maintenance-naïve, 51% were ICS-free. Mean baseline albuterol use was 5.1 puffs/day. In the ITT population, UMEC/VI, UMEC, and VI reduced the risk of a first exacerbation versus PBO by 58, 44, and 39%, respectively (all *p* < 0.05). UMEC/VI provided significant risk reductions versus PBO in all subgroups. VI had no benefit versus PBO in maintenance-naïve, ICS-free, and low rescue use patients and was significantly less effective than UMEC/VI in these subgroups. UMEC had no significant benefit versus PBO in maintenance-naïve and ICS-free patients. All bronchodilators improved FEV_1_ versus PBO, and UMEC/VI significantly improved FEV_1_ versus both monotherapies across all populations studied (*p* < 0.05). All bronchodilators were similarly well tolerated.

**Conclusions:**

Results suggest that UMEC/VI reduces exacerbation risk versus PBO more consistently across medication subgroups than UMEC or VI, particularly in patients with no/low concurrent medication use. Confirmed prospectively, these findings may support first-line use of dual bronchodilation therapy in symptomatic low-risk patients.

**Electronic supplementary material:**

The online version of this article (10.1186/s12931-019-1027-9) contains supplementary material, which is available to authorized users.

## Background

Decreasing the frequency and severity of exacerbations is a key goal of treatment for patients with chronic obstructive pulmonary disease (COPD). However, for patients without a known exacerbation risk, the main treatment goals are to improve symptoms, health-related quality of life, and exercise tolerance [1].

According to the Global initiative for chronic Obstructive Lung Disease (GOLD) 2019 report, the preferred initial maintenance therapies for symptomatic patients with COPD are long-acting bronchodilators [1]. The stepwise treatment paradigm in the GOLD report recommends use of either long-acting β_2_-agonist (LABA) or long-acting muscarinic antagonist (LAMA) alone, followed by escalation to LAMA/LABA combination therapy in patients with persistent symptoms. In patients classified as GOLD 2019 group B with severe breathlessness, LAMA/LABA combination therapy may be used as an initial therapy. As exacerbation risk increases, inhaled corticosteroids (ICS) may also be added to mono- or dual-bronchodilator therapy [1].

Patients with a high level of symptoms (eg, significant dyspnea [baseline modified Medical Research Council {mMRC} score ≥ 2]) yet low risk of exacerbations (mirroring GOLD 2019 group B^1^) are likely to have significant disease burden and increased potential for disease instability and progression [2, 3]. In the 3-year prospective ECLIPSE study, 1 in 4 highly symptomatic, low-risk patients with moderate airflow limitation [4] had ≥ 1 hospitalization during the subsequent 3-year follow-up period [2]. In addition, a large, 3-year study conducted in a Danish national cohort showed increased risk of both respiratory-related mortality and all-cause mortality in patients with an mMRC score ≥ 2 compared with less symptomatic patients. This increased risk was independent of baseline exacerbation risk [3]. These large and robust epidemiology studies confirm that future disease instability is likely in breathless patients including those at low risk of exacerbations.

The lack of disease stability in symptomatic patients may be improved by means of better bronchodilation (ie, improvement in trough forced expiratory volume in 1 s [FEV_1_] above the minimally clinically relevant threshold of 100 mL is predicted to provide clinically relevant reductions in exacerbation risk) [5–7]. LAMA/LABA combination therapy has been shown to provide greater clinically relevant improvements in lung function, as well as greater improvements in dyspnea and health-related quality of life, compared with monotherapy [6, 8–12]. However, the effect of combination bronchodilator therapy in reducing exacerbation risk compared with monotherapy differs between studies, with inconsistent findings [10, 13, 14].

In a meta-analysis of long-acting bronchodilator therapy where COPD exacerbation data were available for 16 randomized control trials (RCTs; 18,224 patients), LAMA/LABA therapy resulted in reduced moderate/severe exacerbation risk compared with placebo (PBO) and LABA, but not LAMA, monotherapy [10]. However, this meta-analysis failed to account for within-class efficacy differences between LAMAs, LABAs and their combinations [15–17], and no adjustment was made for concurrent inhaled respiratory therapy, such as ICS, which may limit generalizability and lead to potential confounding [10]. Indeed, in exacerbation-focused trials comparing LAMA + LABA therapies with the LAMA monotherapy tiotropium (TIO), no clear difference in moderate/severe exacerbation risk was reported [13, 14, 18]. The large DYNAGITO (NCT02296138) [13] and SPARK (NCT01120691) [14] trials allowed widespread continued use of ICS, and analyses in non-ICS users showed no trend for improvement in exacerbation risk with combination therapy. Furthermore, both trials included populations with potentially frequent as-needed use of the short-acting β_2_-agonist (SABA) albuterol at baseline. As SABA use has been shown to correlate with and predict exacerbation risk in COPD [19–21], the supplemental use of a non-randomized bronchodilator may have also increased confounding in these trials. In addition, the protective effects of earlier LAMA/LABA use in lower risk symptomatic patients with COPD has not been adequately explored.

To better understand the role of LAMA/LABA, LAMA, and LABA therapies on exacerbation risk, we aimed to explore the potential clinical impact of concurrent inhaled therapy on exacerbation prevention afforded by different bronchodilator types in symptomatic patients with COPD at low-risk of exacerbations using integrated data from two large 24-week, PBO-controlled bronchodilator trials [22, 23]. Subgroup analyses based on existing maintenance treatment, ICS use and as-needed albuterol use were performed to improve understanding of how these factors may influence changes in lung function and exacerbation prevention for each bronchodilator type versus PBO, and to explore the inter-dependency of lung function improvement and the magnitude of exacerbation prevention.

## Methods

### Study design

This integrated post hoc intent-to-treat (ITT) analysis (GSK study number: 209304) evaluated prospectively collected data from two large 24-week, randomized, double-blind PBO-controlled trials with replicate design (GSK Studies: DB2113361 [NCT01313637; *n* = 1489] [22] and DB2113373 [NCT01313650; *n* = 1532]) [23]. In both studies, symptomatic patients (mMRC dyspnea scale score ≥ 2), ≥ 40 years of age with a diagnosis of moderate-to-very-severe COPD (post-albuterol FEV_1_ ≤ 70% of predicted normal) in accordance with the American Thoracic Society/European Respiratory Society (ATS/ERS) definition [24], with/without a history of exacerbations in the prior year, were randomized (2:3:3:3) to once-daily: PBO, UMEC/VI (62.5/25 μg [NCT01313650] or 125/25 μg [NCT01313637]), UMEC (62.5 μg [NCT01313650] or 125 μg [NCT01313637]), or VI 25 μg, each administered via the ELLIPTA inhaler. Both trials examined trough FEV_1_ on treatment Day 169 as a primary endpoint and prospectively collected moderate and severe exacerbation data.

### Study endpoints

The focus of this post hoc secondary analysis was to examine the time to first moderate/severe COPD exacerbation for each bronchodilator type compared with PBO and to compare change from baseline in trough FEV_1_ during 6-months of therapy. An exacerbation was defined as an acute worsening of symptoms of COPD requiring emergency treatment, hospitalization or use of any additional pharmacotherapy (eg, oral steroids or antibiotics) beyond the study drug or rescue albuterol. An additional endpoint was subjectively-assessed symptom burden measured by the transition dyspnea index (TDI) focal score. The influence of concomitant ICS or rescue medication (albuterol) and their impact on these outcomes was also explored. Safety endpoints included incidence of adverse events (AEs).

### Data analyses

Post hoc analyses were conducted for the integrated ITT population from both studies and for each concurrent medication subgroup separately. Treatment groups were combined regardless of the two different UMEC doses used alone or in combination with VI; however, study NCT01313637 tested UMEC and UMEC/VI at only investigational doses [22]. The ITT population was comprised of all patients randomized to treatment who received ≥ 1 dose of randomized study medication during the treatment period. Treatment with ICS was permitted at baseline, and post-randomization at a constant dose corresponding to that used within the ICS containing regimen at screening. Concurrent medication subgroups were segmented by patient treatment status at screening and baseline: maintenance-naïve (no COPD medication except short-acting bronchodilators used as rescue medication recorded in the 30 days prior to screening) or on maintenance therapy, ICS-free (at the time of the screening visit) or ICS-treated, and low or high rescue albuterol use at baseline based on splitting the population by median run-in use (< 3.6 or ≥ 3.6 puffs/day; corresponding to < 25 or ≥ 25 puffs/week). The treatment comparisons performed were UMEC/VI, UMEC and VI versus PBO, and UMEC/VI versus UMEC or VI.

Trough FEV_1_ was assessed on Days 2, 28, 56, 84, 112, 168 and 169 using a mixed model repeated measures (MMRM) analysis, with covariates for baseline FEV_1_, smoking status, Day, center group, treatment, day by baseline interaction and day by treatment interaction. TDI was assessed on Days 28, 84, and 168 using a MMRM analysis with covariates for study, treatment, smoking status at screening, baseline dyspnea index (BDI) focal score, day, geographical region, day by BDI focal score and day by treatment interaction. Least squares (LS) mean change from baseline values and their associated standard errors (SE) were generated for each treatment group; estimated treatment difference and corresponding 95% confidence intervals (CI) and *p*-values were also reported for the ITT population and each concurrent medication subgroup.

The percent change from baseline in trough FEV_1_ for each patient expressed as a ratio was also analyzed using MMRM analysis with a response of log (trough FEV_1_/baseline FEV_1_) with log (baseline FEV_1_), smoking status, Day, center group, treatment, day by log (baseline FEV_1_) interaction, and day by treatment interaction as covariates. Results were back transformed to provide point estimates. Risk of a first moderate/severe exacerbation was assessed using Kaplan–Meier (KM) and Cox proportional hazards model for the ITT population and for each medication subgroup separately, with covariates for study, treatment, smoking status at screening and center group. Hazard ratios (HR) and 95% CIs were reported. As this was a PBO-controlled blinded study, patients were withdrawn from the study following a first exacerbation on any treatment arm (active or PBO), to allow for more appropriate treatment. Safety analyses assessed AE data and were conducted for the integrated ITT population; safety data were analyzed descriptively.

## Results

### Study population

Of the 3021 patients included in the integrated ITT population, 816, 825, 825, and 555 received UMEC/VI, UMEC, VI, or PBO, respectively; baseline characteristics were comparable across all treatment groups (Table 1). At study entry, the mean age in the ITT population was 63 years, 68% were male and approximately one-third (36%) had a moderate (26%) or severe (10%) exacerbation in the previous year (Table 1). Across all treatment arms, one-third (33%) of patients were maintenance treatment-naïve at screening and approximately half (51%) were using ICS. Mean baseline albuterol use for the ITT population was 5.1 puffs/day, with equal proportions of the ITT population being arbitrarily divided into the low and high albuterol use medication subgroups (according to overall median SABA use across all treatment groups at baseline [ie, < or ≥ 3.6 puffs/day corresponding to < or ≥ 25 puffs/week]).

**Table 1:** Patient demographics (integrated ITT population)

	Total (*N* = 3021)	UMEC/VI (*n* = 816)	UMEC (*n* = 825)	VI (*n* = 825)	PBO (*n* = 555)
Age, years, mean (SD)	63.0 (8.7)	63.3 (8.4)	63.5 (8.8)	62.7 (8.7)	62.2 (8.8)
Male, n (%)	2057 (68)	569 (70)	568 (69)	550 (67)	370 (67)
BMI, kg/m^2^, mean (SD)	26.7 (5.8)	26.9 (5.6)	26.4 (5.7)	26.9 (6.0)	26.7 (6.0)
Current smoker, n (%)	1528 (51)	403 (49)	423 (51)	409 (50)	293 (53)
Smoking pack years^a^, mean (SD)	45.1 (24.8)	45.9 (25.7)	45.4 (25.3)	43.8 (23.2)	45.4 (25.3)
Pre-bronchodilator FEV_1_, mL, mean (SD)^b^	1258 (486)	1272 (503)	1254 (484)	1264 (490)	1234 (460)
Post-bronchodilator % predicted FEV_1_, mean (SD)^c^	47.8 (12.9)	47.8 (12.9)	47.8 (12.9)	48.4 (13.0)	47.1 (12.6)
GOLD stage III-IV, FEV_1_ < 50% predicted, n (%)^c^	1605 (53)	435 (54)	438 (53)	418 (51)	314 (57)
Exacerbation history, n (%)^d^					
≥ 1 moderate exacerbation^e^	780 (26)	210 (26)	211 (26)	214 (26)	145 (26)
≥ 1 severe exacerbation^f^	302 (10)	72 (9)	82 (10)	93 (11)	55 (10)
Baseline mMRC dyspnea score, mean (SD)	2.4 (0.5)	2.4 (0.5)	2.4 (0.6)	2.3 (0.6)	2.4 (0.5)
Baseline albuterol use puffs/day, mean (SD)^g,h^	5.1 (5.4)	4.8 (5.1)	5.1 (5.4)	5.2 (5.4)	5.3 (5.9)
Maintenance-naïve, n (%)^i^					
Yes	984 (33)	285 (35)	245 (30)	272 (33)	182 (33)
No	2037 (67)	531 (65)	580 (70)	553 (67)	373 (67)
ICS-free, n (%)					
Yes	1543 (51)	428 (52)	413 (50)	422 (51)	280 (50)
No	1478 (49)	388 (48)	412 (50)	403 (49)	275 (50)
Baseline albuterol use, n (%)^g,j^					
High	1490 (50)	386 (48)	405 (50)	411 (51)	288 (53)
Low	1488 (50)	420 (52)	408 (50)	401 (49)	259 (47)

^a^Smoking pack years = (number of cigarettes smoked per day/20) x number of years smoked. ^b^Total: *n* = 3015; UMEC/VI: *n* = 815; UMEC: *n* = 824; VI: *n* = 822; PBO: *n* = 554. ^c^Total: *n* = 3012; UMEC/VI: *n* = 813; UMEC: *n* = 823; VI: *n* = 822; PBO: *n* = 554. ^d^Number of COPD exacerbations reported in the 12-months prior to screening. ^e^Required oral/systemic corticosteroids and/or antibiotics. ^f^Required hospitalization. ^g^Total: *n* = 2978; UMEC/VI: *n* = 806; UMEC: *n* = 813; VI: *n* = 812; PBO: *n* = 547. ^h^Baseline rescue medication use was calculated for patients with non-missing values for at least half the days within the baseline period. ^i^Prior to run-in. ^j^Mean puffs/day at baseline ≥ 3.60 (high) or < 3.60 (low); 3.60 is the median value of puffs of rescue medication per day at baseline across all treatment groups

*BMI* body mass index, *COPD* chronic obstructive pulmonary disease, *FEV*_*1*_ forced expiratory volume in 1 s, *GOLD* Global initiative for chronic Obstructive Lung Disease, *ICS* inhaled corticosteroid, *ITT* intent-to-treat, *mMRC* modified medical research council, *PBO* placebo, *SD* standard deviation, *UMEC* umeclidinium, *VI* vilanterol

### Exacerbation risk reduction

In the full ITT population, 259 (9%) patients experienced ≥ 1 on-treatment moderate/severe exacerbation across all treatment arms during the treatment period. Overall, 73 (13%) patients treated with PBO experienced a first exacerbation, although this varied by baseline concurrent medication use; the lowest incidence of exacerbations being in the maintenance-naïve, ICS-free, and low rescue use subgroups (9–10% incidence), and the highest incidence of exacerbations being in the on-maintenance, ICS-treated, and high rescue use subgroups (15–17%).

**Fig. 1:**
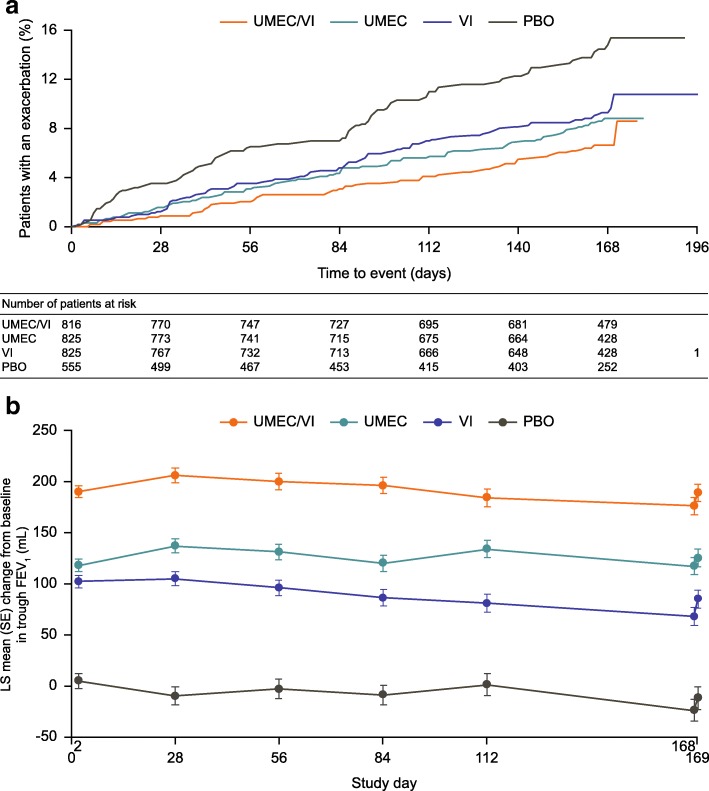
Time to first exacerbation (**a**) and trough FEV_1_ profile (**b**) (ITT population). *CI*, confidence interval; *FEV*_*1*_, forced expiratory volume in 1 s; *ITT*, intent-to-treat; *LS*, least squares; *PBO*, placebo; *SE*, standard error; *UMEC*, umeclidinium; *VI*, vilanterol

At any given timepoint, the probability of patients in the ITT population experiencing an exacerbation was lower for patients receiving UMEC/VI, UMEC, or VI than for those receiving PBO (Fig. 1a). UMEC/VI, UMEC, and VI reduced the risk of a first exacerbation versus PBO by 58, 44, and 39%, respectively (all *p* < 0.05; Table 2).

**Table 2:** Percentage risk reduction (95% CI) for a first moderate/severe exacerbation

	UMEC/VI vs PBO	UMEC vs PBO	VI vs PBO	UMEC/VI vs UMEC	UMEC/VI vs VI
Patient group/subgroup					
ITT population (*N* = 3021)	**58 (40, 71)** ***p*** **< 0.001**	**44 (22, 60)** ***p*** **< 0.001**	**39 (15, 56)** ***p*** **= 0.003**	24 (−9, 48) *p* = 0.136	**31 (2, 52)** ***p*** **= 0.041**
Patient subgroups					
Maintenance-naïve (*n* = 984)	**62 (15, 83)** ***p*** **= 0.018**	22 (−55, 61) *p* = 0.475	4 (−82, 49) *p* = 0.904	51 (−8, 77) *p* = 0.076	**60 (16, 81)** ***p*** **= 0.016**
On maintenance (*n* = 2037)	**56 (35, 71)** ***p*** **< 0.001**	**50 (27, 66)** ***p*** **< 0.001**	**48 (24, 65)** ***p*** **< 0.001**	12 (−33, 42) *p* = 0.540	16 (−27, 45) *p* = 0.408
ICS-free (*n* = 1543)	**62 (30, 80)** ***p*** **= 0.002**	41 (−3, 66) *p* = 0.063	15 (−41, 49) *p* = 0.520	36 (−20, 66) *p* = 0.166	**55 (19, 75)** ***p*** **= 0.008**
ICS-treated (*n* = 1478)	**55 (30, 71)** ***p*** **< 0.001**	**46 (19, 65)** ***p*** **= 0.003**	**51 (25, 68)** ***p*** **= 0.001**	15 (−33, 46) *p* = 0.470	7 (−48, 42) *p* = 0.753
Low rescue use (*n* = 1488)	**71 (43, 85)** ***p*** **< 0.001**	**51 (12, 72)** ***p*** **= 0.018**	32 (−16, 61) *p* = 0.153	41 (−18, 70) *p* = 0.135	**57 (17, 78)** ***p*** **= 0.012**
High rescue use (*n* = 1490)	**49 (22, 67)** ***p*** **= 0.002**	**43 (13, 62)** ***p*** **= 0.009**	**45 (15, 64)** ***p*** **= 0.006**	12 (−38, 44) *p* = 0.580	9 (−44, 42) *p* = 0.699

Risk of a first moderate/severe exacerbation was assessed using a Cox proportional hazards model for all medication subgroups separately, with covariates for study, treatment, smoking status at screening and center group. Bold values indicate statistical significance (*p* < 0.05)

Low rescue use: < 3.6 puffs/day; high rescue use: ≥ 3.6 puffs/day

*CI* confidence interval, *ICS* inhaled corticosteroid, *ITT* intent-to-treat, *LS* least squares, *PBO* placebo, *UMEC* umeclidinium, *VI* vilanterol

**Fig. 2:**
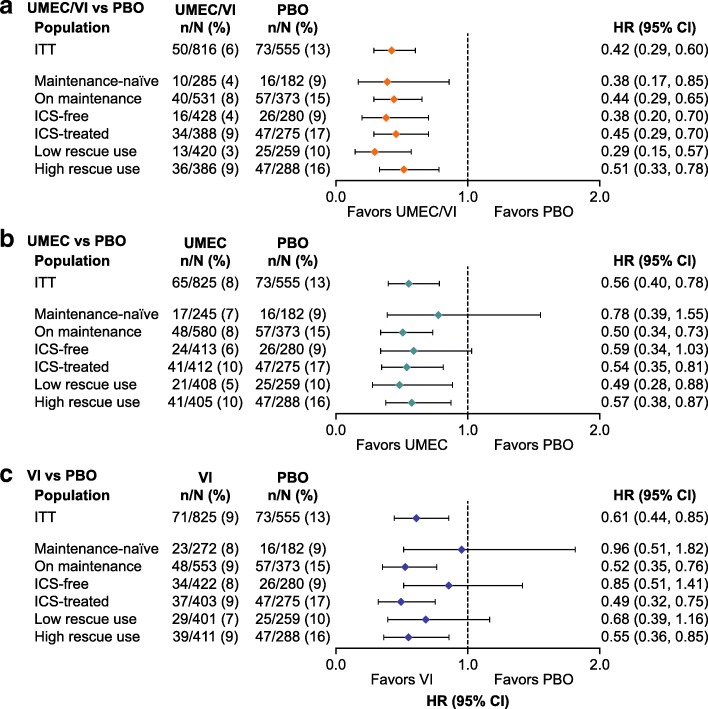
Forest plot of HR (95% CI) for a first moderate/severe exacerbation: UMEC/VI versus PBO (**a**), UMEC versus PBO (**b**), and VI versus PBO (**c**). Risk of a first moderate/severe exacerbation was assessed using a Cox proportional hazards model for all medication subgroups separately, with covariates for study, treatment, smoking status at screening and center group. *CI*, confidence interval; *HR*, hazard ratio; *ICS*, inhaled corticosteroid; *ITT*, intent-to-treat; *PBO*, placebo; *UMEC*, umeclidinium; *VI*, vilanterol

UMEC/VI was the only bronchodilator to provide a significant risk reduction versus PBO in all concurrent medication subgroups (49–71% risk reduction; *p* < 0.05), including both the maintenance-naïve and ICS-free subgroups (Table 2 and Fig. 2). Across the six medication subgroups, there was a lack of consistency in exacerbation prevention for both monotherapies versus PBO, which was most apparent for VI (4–51%). The corresponding risk reduction for UMEC versus PBO ranged from 22 to 51% across the medication subgroups (Table 2 and Fig. 2).

In the pairwise comparisons between active therapies for the ITT population, UMEC/VI delivered a statistically significant benefit over VI in reducing the risk of a first exacerbation (31%; *p* = 0.041). This was predominantly driven by greater reductions in exacerbation risk in the maintenance-naïve, ICS-free and low rescue use medication subgroups (55–60% risk reduction versus VI; *p* < 0.05; Table 2). No statistically significant reductions in exacerbation risk were observed for UMEC/VI versus UMEC monotherapy in the ITT population or any medication subgroup.

### Change from baseline in trough FEV_1_

At any given timepoint, significantly greater improvements in trough FEV_1_ were seen for patients in the ITT population receiving UMEC/VI, UMEC, or VI compared with patients receiving PBO (all *p* < 0.001; Fig. 1b). Similar results were also observed across all medication subgroups, for both absolute and percent change from baseline in trough FEV_1_ (all *p* ≤ 0.001; Day 169 data presented in Table 3). UMEC/VI also provided greater improvements in trough FEV_1_ (absolute or percent change from baseline) versus UMEC and VI monotherapies in the ITT population and all medication subgroups at Day 169 (all *p* < 0.05; Table 3).

**Table 3:** LS mean (95% CI) change from baseline in trough FEV_1_ at Day 169

Patient group/subgroup	UMEC/VI vs PBO		UMEC vs PBO		VI vs PBO		UMEC/VI vs UMEC		UMEC/VI vs VI	
	Δ mL	Δ %	Δ mL	Δ %	Δ mL	Δ %	Δ mL	Δ %	Δ mL	Δ %
ITT population (*N* = 3021)	200	17.8	136	11.8	96	8.5	64	5.4	104	8.6
	(173, 228)	(15.4, 20.3)	(109, 164)	(9.5, 14.1)	(69, 124)	(6.2, 10.7)	(40, 88)	(3.5, 7.4)	(80, 128)	(6.7, 10.6)
	*p* < 0.001	*p* < 0.001	*p* < 0.001	*p* < 0.001	*p* < 0.001	*p* < 0.001	*p* < 0.001	*p* < 0.001	*p* < 0.001	*p* < 0.001
Patient subgroups										
Maintenance-naïve	209	17.2	149	12.3	137	11.6	60	4.3	72	5.1
(*n* = 984)	(159, 260)	(13.1, 21.5)	(97, 202)	(8.3, 16.6)	(85, 189)	(7.6, 15.7)	(14, 106)	(1.0, 7.7)	(27, 117)	(1.8, 8.4)
	*p* < 0.001	*p* < 0.001	*p* < 0.001	*p* < 0.001	*p* < 0.001	*p* < 0.001	*p* = 0.011	*p* = 0.010	*p* = 0.002	*p* = 0.002
On maintenance	195	18.1	131	11.6	75	6.9	63	5.8	120	10.5
(*n* = 2037)	(162, 227)	(15.2, 21.2)	(99, 163)	(8.8, 14.4)	(42, 107)	(4.2, 9.6)	(35, 91)	(3.5, 8.2)	(91, 148)	(8.1, 13.0)
	*p* < 0.001	*p* < 0.001	*p* < 0.001	*p* < 0.001	*p* < 0.001	*p* < 0.001	*p* < 0.001	*p* < 0.001	*p* < 0.001	*p* < 0.001
ICS-free (*n* = 1543)	204	17.7	150	12.9	127	11.1	54	4.2	76	5.9
	(164, 243)	(14.4, 21.1)	(110, 190)	(9.7, 16.2)	(88, 167)	(8.0, 14.4)	(19, 88)	(1.6, 6.8)	(41, 111)	(3.3, 8.6)
	*p* < 0.001	*p* < 0.001	*p* < 0.001	*p* < 0.001	*p* < 0.001	*p* < 0.001	*p* = 0.003	*p* = 0.001	*p* < 0.001	*p* < 0.001
ICS-treated (*n* = 1478)	198	18.1	124	10.8	64	5.8	74	6.6	134	11.7
	(160, 236)	(14.6, 21.7)	(86, 162)	(7.5, 14.1)	(26, 102)	(2.6, 9.0)	(41, 107)	(3.9, 9.5)	(101, 168)	(8.8, 14.7)
	*p* < 0.001	*p* < 0.001	*p* < 0.001	*p* < 0.001	*p* = 0.001	*p* < 0.001	*p* < 0.001	*p* < 0.001	*p* < 0.001	*p* < 0.001
Low rescue use	206	17.2	158	13.0	101	8.3	48	3.7	105	8.2
(*n* = 1488)	(169, 243)	(14.1, 20.4)	(121, 195)	(10.0, 16.1)	(64, 139)	(5.4, 11.3)	(16, 80)	(1.3, 6.1)	(72, 137)	(5.7, 10.7)
	*p* < 0.001	*p* < 0.001	*p* < 0.001	*p* < 0.001	*p* < 0.001	*p* < 0.001	*p* = 0.003	*p* = 0.002	*p* < 0.001	*p* < 0.001
High rescue use	194	18.4	113	10.3	88	8.3	81	7.3	106	9.4
(*n* = 1490)	(153, 235)	(14.7, 22.3)	(72, 154)	(6.8, 13.9)	(47, 129)	(4.9, 11.8)	(44, 118)	(4.3, 10.5)	(69, 142)	(6.3, 12.5)
	*p* < 0.001	*p* < 0.001	*p* < 0.001	*p* < 0.001	*p* < 0.001	*p* < 0.001	*p* < 0.001	*p* < 0.001	*p* < 0.001	*p* < 0.001

Δ, pairwise treatment comparison; low rescue use: < 3.6 puffs/day; high rescue use: ≥ 3.6 puffs/day

*BL* baseline, *CI* confidence interval, *ICS* inhaled corticosteroid, *ITT* intent-to-treat, *LS* least squares, *PBO* placebo, *UMEC* umeclidinium, *VI* vilanterol

### Lung function improvements versus exacerbation risk reduction

**Fig. 3:**
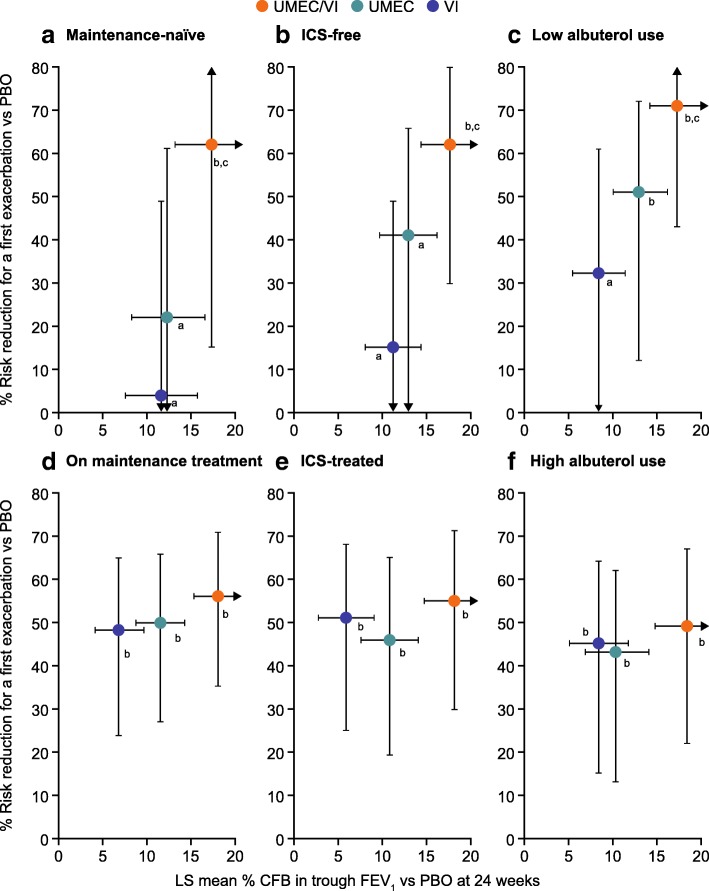
Trough FEV_1_ CFB (active treatment vs PBO) versus exacerbation risk reduction by baseline medication subgroup: Maintenance-naive (**a**), ICS-free (**b**), Low albuterol use (**c**), On maintenance treatment (**d**), ICS-treated (**e**), and High albuterol use (**f**). ^a^ *p* < 0.001 versus PBO for improvement in trough FEV_1_ and no significant impact on exacerbation risk versus PBO. ^b^ *p* < 0.001 versus PBO for improvement in trough FEV_1_ and significant (*p* < 0.05) reduction versus PBO in risk of a first exacerbation. ^c^ *p* < 0.05 versus VI for improvement in FEV_1_ and reduction in risk of a first exacerbation within subgroup. All pairwise comparisons represent exacerbation risk ratios (95% CI) plotted against LS means (95% CI) CFB in trough FEV_1_ for the comparison of each active treatment versus placebo. *CFB*, change from baseline; *FEV*_*1*_, forced expiratory volume in 1 s; *ICS*, inhaled corticosteroid; *LS*, least squares, *PBO*, placebo; *UMEC*, umeclidinium; *V*I, vilanterol

For each baseline medication subgroup, the LS mean change from baseline versus placebo in trough FEV_1_ at Day 169 was plotted against the percent reduction in exacerbation risk versus PBO, for each active therapy (Fig. 3). In the low use of concurrent medication subgroups (maintenance-naïve, ICS-free, low albuterol use), increased bronchodilation with UMEC/VI provided on average both higher percent increases in trough FEV_1_ and increased reductions in exacerbation risk compared with either UMEC or VI alone (Fig. 3a–c). In the high use of concurrent medication subgroups (on maintenance treatment, ICS-treated, high albuterol use) similar percentage increases in trough FEV_1_ compared with placebo were observed as in the low concurrent medication subgroups. However, in contrast to the low concurrent medication subgroups, minimal differences in exacerbation protection were observed between the three active therapies (Fig. 3d–f).

### Symptoms

At all timepoints, patients in the ITT population experienced statistically significant improvements in dyspnea with active therapies versus PBO (*p* < 0.001 at all timepoints) and with UMEC/VI versus both monotherapies (*p* < 0.01 at all timepoints; Additional file 1). UMEC/VI was the only active therapy to demonstrate clinically relevant improvements in dyspnea (≥ 1.0 unit in TDI) versus PBO, with improvements of > 1.0 unit observed in the ITT population, and in the on-maintenance, ICS-treated, and low albuterol use subgroups (Additional file 2). The greatest differentiation between UMEC/VI and the monotherapies was seen in the low albuterol use subgroup, and the lowest differentiation was seen in the high albuterol use subgroup (Additional file 2).

### Safety

Overall, all bronchodilators were well tolerated with similar incidence of AEs compared with PBO (UMEC/VI: 423 [52%]; UMEC: 433 [52%]; VI: 419 [51%]; PBO: 264 [48%]; Additional file 3); safety profiles were similar for both dual therapy and monotherapies. The most common AEs across all assessed treatments included nasopharyngitis (8–11% incidence in any treatment group), headache (8–10%), and cough (4%). Serious AEs occurred in 5–6% patients, and AEs leading to treatment discontinuation or study withdrawal occurred in 5–7% of patients across all treatment groups. Fatal SAEs occurred in < 1% of patients in each treatment arm (Additional file 3). Detailed safety results are available in the original studies [22, 23].

## Discussion

This post hoc integrated analysis demonstrated a statistically significant improvement in trough FEV_1_ and reduction in COPD exacerbation risk in the integrated ITT population with all bronchodilators studied compared with PBO. The magnitude of improvement for trough FEV_1_ and risk of a first exacerbation favored UMEC/VI compared with both monotherapies, although the incremental benefit on exacerbation prevention in the ITT population with UMEC/VI was only significant versus VI. Exacerbation incidence was higher in patients receiving PBO in the on-maintenance, ICS-treated and high albuterol use subgroups; however, the ability to discriminate between dual- and monotherapies on the prevention of exacerbations in these subgroups was diminished. By contrast, the maintenance-naïve, ICS-free, and low albuterol subgroups showed greater exacerbation prevention with UMEC/VI versus UMEC and VI monotherapies. Unlike UMEC/VI, neither of the monotherapies provided a statistically significant reduction in exacerbation risk versus PBO in the maintenance-naïve and ICS-free medication subgroups. For both UMEC and VI monotherapies, the improvement in lung function achieved versus PBO was broadly similar across the six medication subgroups, whereas the corresponding magnitude of exacerbation prevention fluctuated widely. In contrast, consistent and similar levels of efficacy on lung function and exacerbation prevention were observed in all subgroups with UMEC/VI compared with PBO.

The statistically significant, but weak association between increasing bronchodilation and increasing prevention of exacerbations has been highlighted and debated in several review articles [5–7]. Our subgroup analysis indicates that while increased bronchodilation with UMEC/VI provided additional lung function benefits versus UMEC or VI alone in the high use of concurrent medication subgroups (on maintenance treatment, ICS-treated, high albuterol use), between-treatment differences in terms of exacerbation reduction were not similarly observed. In contrast, in the subgroups of patients with minimal exposure to prior maintenance and as-needed medications, increased bronchodilation was generally associated with increased reduction in exacerbation risk compared with placebo. These data suggest that the ability to predict increased exacerbation prevention with increasing bronchodilation may be limited in patients not new to maintenance therapy, receiving concomitant ICS and/or over-relying on rescue SABA. These results offer a potential explanation for the failure to obtain clinically meaningful and statistically significant benefits on moderate/severe exacerbation outcomes with LAMA/LABA combinations (indacaterol/glycopyrronium [IND/GLY] and TIO/olodaterol [OLO]) versus TIO in two of the largest prospective exacerbation trials comparing these bronchodilator classes to date [13, 14]. These RCTs were performed in patients with an exacerbation history in the past year, where approximately 70–75% of patients were using ICS therapy at baseline, and over-reliance on albuterol use was commonplace, with mean baseline levels in the SPARK trial similar to those in the current study [13, 14].

Over-reliance on albuterol use is a predictor of short- and long-term exacerbation risk in COPD and a marker for the need for improved maintenance therapy [19, 20]. Reducing as-needed albuterol use is also well correlated with improved health-related quality of life and reduced annual exacerbation rates [21]. These findings suggest that over-reliance on as-needed albuterol should act as a red flag in patients with COPD, highlighting the symptomatic burden of disease and potential inadequacy of their current care. In this analysis, patients were divided into two albuterol use subgroups based on median baseline use. Greater differentiation in the levels of exacerbation prevention and relief of dyspnea, assessed subjectively by TDI focal score, with dual therapy versus monotherapy was seen in low albuterol users. A positive relationship between change in trough FEV_1_ and exacerbation prevention was also observed in this subgroup; however, this relationship was not observed in the high albuterol users. Trough FEV_1_ was measured at clinic visits, with a 4-h minimum washout period for albuterol use. Consequently, the impact of albuterol on top of maintenance bronchodilation or PBO was not captured. The potential for lung function measured at the clinic to be confounded by as-needed albuterol use at home may reduce the potential for group mean data to show a correlation between clinic lung function assessments and exacerbation prevention, particularly for high albuterol users.

This study provides functional insights on the different pharmacological properties of the two main bronchodilator classes, which act on airway smooth muscle cells to induce relaxation via activation of the β_2_-adrenoceptors (LABAs) or via antagonism of acetylcholine at muscarinic M3 receptors (LAMAs), and the potential for cross-talk between these actions at a cellular level [25]. It is widely accepted that the mechanism of action by which both LAMA and LABA bronchodilator types prevent exacerbations is via improving airway mechanics, reducing both airflow obstruction and hyperinflation to ease breathing and decrease symptom severity and variability [26, 27]. Long-acting bronchodilators also have the potential to impact mucus secretions and mucocilliary clearance, and may have impacts on airway smooth muscle growth and airway fibrosis as well as potential anti-inflammatory effects on airway smooth muscle cells; however, these putative mechanisms of action are of unknown clinical importance in preventing exacerbations [26–28]. Consequently, maximal bronchodilation and lung deflation in symptomatic patients are likely to deliver both immediate symptom gain and improved COPD stability with increased protection from future exacerbations.

The current analysis found a large divergence in the overall prevention of exacerbations observed with VI compared with PBO depending on concurrent ICS use, whereas no similar divergence was observed with UMEC. This suggests that the prevention of exacerbation afforded by a LABA, but not a LAMA, may be blunted without the co-administration of corticosteroids. Our findings corroborate those observed in two large exacerbation trials comparing LAMA and LABA where the benefit of TIO (vs either salmeterol twice daily or IND once-daily) on reducing the rate of exacerbations was more evident in ICS-free patients [29, 30]. In the current study, UMEC/VI appeared to provide additional benefits in terms of exacerbation prevention over UMEC in both ICS-free and maintenance-naïve patients, suggesting incremental effects in lower risk patients. This observation may be important as exacerbation trials in patients with advanced COPD have indicated no additional reduction in moderate/severe exacerbations with LAMA + LABA versus the LAMA TIO in the ITT population and particularly in ICS-free patients [13, 14, 18]. Taken together, these data may support a role for LAMA/LABA combinations versus LAMA in preventing exacerbations in symptomatic patients with lower exacerbation risk.

The population investigated in this study mirrored a GOLD 2019 group B population (high symptoms and low risk of exacerbations), with analyses focusing on the prevention of exacerbations afforded by all three classes of long-acting bronchodilator given once-daily using identical inhalers. Well-balanced numbers of patients for each bronchodilator type were obtained across all concurrent medication subgroups, allowing the performance of each bronchodilator to be examined in light of adherence or non-adherence to current GOLD strategy (ie, ahead of or in addition to ICS treatment, respectively) [1]. Our findings in the maintenance-naïve and ICS-free medication subgroups suggest that exacerbation prevention was more likely with LAMA/LABA therapy than either monotherapy. These data may therefore strengthen the clinical rationale for first-line LAMA/LABA use in symptomatic patients. Previous post hoc meta-analyses have shown that LAMA/LABAs are well tolerated compared with monotherapy [10] and can improve lung function and symptoms compared with LAMA therapy in maintenance-naïve patients [31, 32]. UMEC/VI has also demonstrated significant improvements in lung function and significant reductions in SABA use versus once-daily TIO/OLO, in symptomatic patients who were maintenance-naïve at study entry [33]. However, given the lack of prospective data generated for LAMA/LABAs versus LAMAs on symptoms and exacerbation outcomes in ICS-free and maintenance-naïve patients, further prospective RCTs are warranted to better inform on the appropriate positioning of the LAMA/LABA class. This is particularly appropriate given the failure of LAMA + LABA combinations to improve exacerbation risk compared with TIO in patients with more advanced COPD at high risk of exacerbations [13, 14, 18].

This study is not without limitations. Although large and replicate in design, both RCTs pooled in this analysis focused on spirometry, and although the overall population was large enough to detect treatment differences in lung function between the active regimens, it was not large enough to examine exacerbation endpoints between the active therapies, particularly in the smallest maintenance-naïve population. Consequently, inference on the size of exacerbation risk reduction with the three different bronchodilator types was drawn primarily from their comparisons with PBO with less weight placed on pairwise active comparisons. It is notable that one of two PBO-controlled RCTs used an unlicensed dose of UMEC alone and in combination with VI [22]; however, the efficacy and safety of the higher and lower UMEC doses in the two RCTs was similar [22, 23]. Finally, as this was a PBO-controlled trial, for patient safety withdrawal followed a first moderate/severe exacerbation to allow active therapy to be initiated. As a result, a KM risk-based survival analysis was employed to examine prevention of exacerbations as no assessment of exacerbation rate was possible. The choice of a time-to-first KM analysis was appropriate as neither of the studies included here enriched the population for patients with an exacerbation history, and very few patients would be likely to suffer recurrent events in the 6-month observation period.

## Conclusion

This post hoc analysis highlights that improving bronchodilation with once-daily UMEC/VI, UMEC and VI provides greater exacerbation prevention compared with PBO in symptomatic, low-risk patients. Unlike either monotherapy, UMEC/VI combination therapy provided significant prevention of exacerbations in both maintenance-naïve and ICS-free patients compared with PBO. The low concurrent medication subgroups also showed a positive relationship between maximizing lung function and exacerbation risk reduction. These results suggest that symptomatic, low-risk patients not already receiving a maintenance therapy may be ideal candidates for LAMA/LABA therapy. Future prospective studies are now needed to explore short-term symptom improvement and risks of deterioration with early dual- and mono-bronchodilator therapy in these patient populations.

## Additional files

Additional file 1: TDI focal score profile (ITT population). (DOCX 78 kb)

Additional file 2: Forest plot of TDI focal score at Day 168, active treatments versus PBO. (DOCX 101 kb)

Additional file 3: Safety endpoints. (DOCX 18 kb)

## Abbreviations

AE: Adverse events
BDI: Baseline dyspnea index
BMI: Body mass index
CFB: Change from baseline
CI: Confidence interval
COPD: Chronic Obstructive Pulmonary Disease
FEV_1_: Forced expiratory volume in 1 s
GLY: Glycopyrronium
GOLD: Global initiative for chronic Obstructive Lung Disease
ICS: Inhaled corticosteroids
IND: Indacaterol
ITT: Intent-to-treat
KM: Kaplan–Meier
LABA: Long-acting β_2_-agonist
LAMA: Long-acting muscarinic antagonist
LS: Least squares
mMRC: Modified medical research council
OLO: Olodaterol
PBO: Placebo
RCT: Randomized control trial
SABA: Short-acting β_2_-agonist
SAEs: Serious adverse events
SD: Standard deviation
SE: Standard error
TDI: Transition dyspnea index
TIO: Tiotropium
UMEC: Umeclidinium
VI: Vilanterol
