# Supplementary material for: Impact of prior and concurrent medication on exacerbation risk with long-acting bronchodilators in chronic obstructive pulmonary disease: a post hoc analysis
Source: Respir Res. 2019 Mar 26;20:60. doi: 10.1186/s12931-019-1027-9 (PMC6434823; doi:10.1186/s12931-019-1027-9)
Supplement: Supplementary file 1 — TDI focal score profile (ITT population). (DOCX 78 kb) [file 12931_2019_1027_MOESM1_ESM.docx]

**Additional file 1: TDI focal score profile (ITT population)**


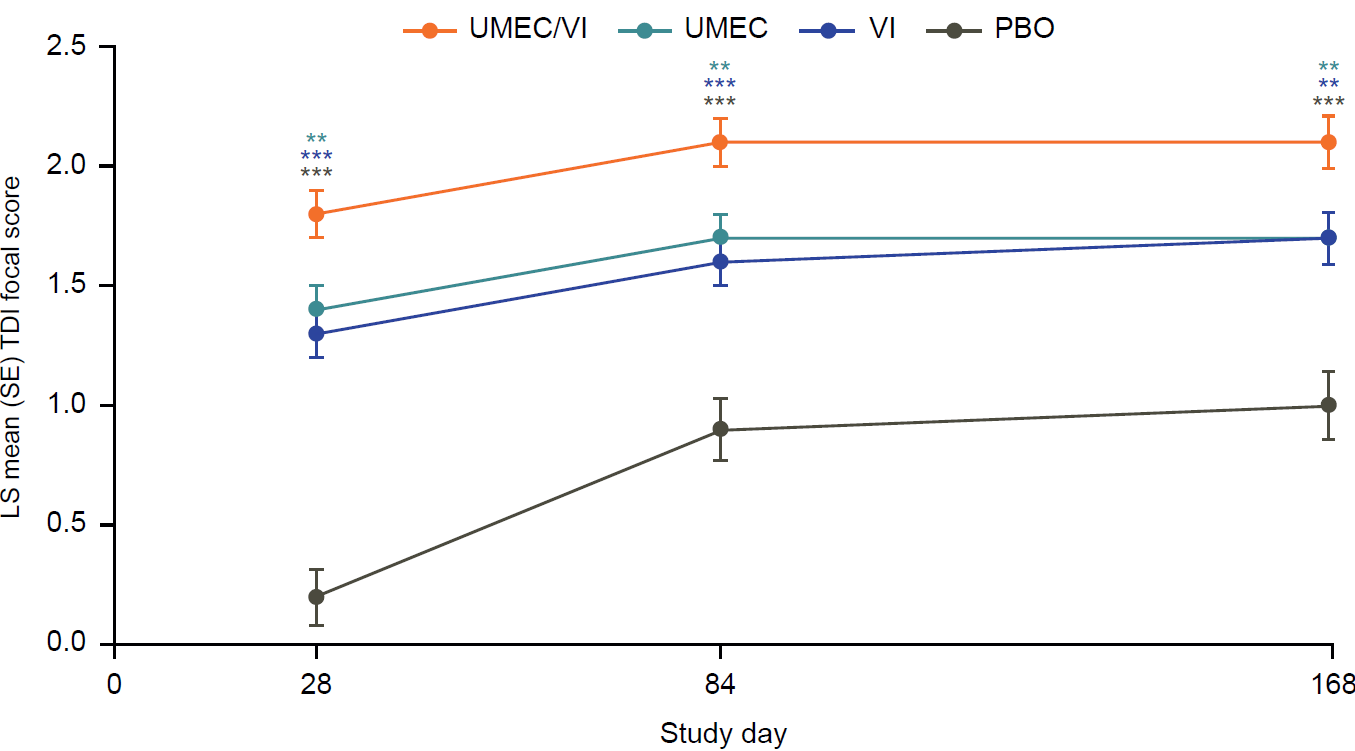


#### p‑values reported indicate significant differences for UMEC/VI versus all three comparators: **p<0.01, ***p<0.001

#### ITT, intent-to-treat; LS, least squares; PBO, placebo; SE, standard error; TDI, transition dyspnea index; UMEC, umeclidinium; VI, vilanterol
