# Supplementary material for: Impact of prior and concurrent medication on exacerbation risk with long-acting bronchodilators in chronic obstructive pulmonary disease: a post hoc analysis
Source: Respir Res. 2019 Mar 26;20:60. doi: 10.1186/s12931-019-1027-9 (PMC6434823; doi:10.1186/s12931-019-1027-9)
Supplement: Supplementary file 2 — Forest plot of TDI focal score at Day 168, active treatments versus PBO. (DOCX 101 kb) [file 12931_2019_1027_MOESM2_ESM.docx]

**Additional file 2: Forest plot of TDI focal score at Day 168, active treatments versus PBO**


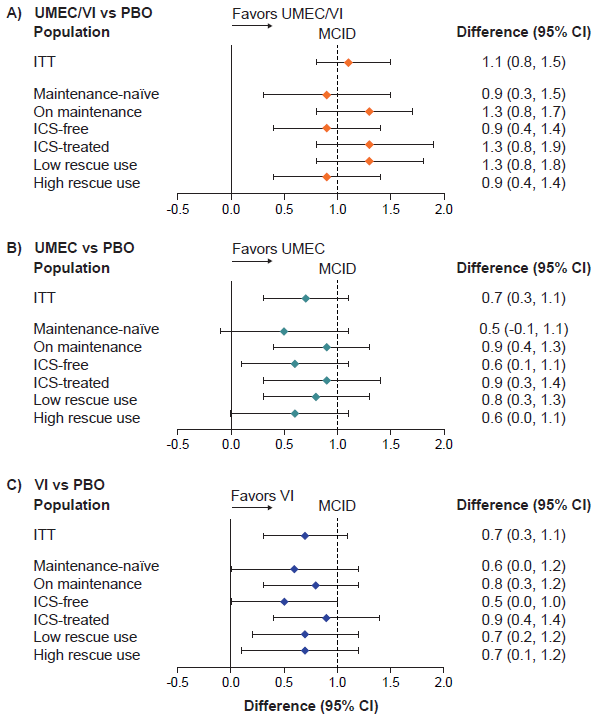


#### CI, confidence interval; ICS, inhaled corticosteroid; ITT, intent-to-treat; MCID, minimal clinically important difference; PBO, placebo; TDI, transition dyspnea index; UMEC, umeclidinium; VI, vilanterol
