# Supplementary material for: Impact of prior and concurrent medication on exacerbation risk with long-acting bronchodilators in chronic obstructive pulmonary disease: a post hoc analysis
Source: Respir Res. 2019 Mar 26;20:60. doi: 10.1186/s12931-019-1027-9 (PMC6434823; doi:10.1186/s12931-019-1027-9)
Supplement: Supplementary file 3 — Safety endpoints. (DOCX 18 kb) [file 12931_2019_1027_MOESM3_ESM.docx]

**Additional File 3: Safety endpoints**

|  | **UMEC/VI**  **(N=816)** | **UMEC**  **(N=825)** | **VI**  **(N=825)** | **PBO**  **(N=555)** |
| --- | --- | --- | --- | --- |
| **AEs** | 423 (52) | 433 (52) | 419 (51) | 264 (48) |
| **SAEs** | 44 (5) | 49 (6) | 44 (5) | 26 (5) |
| **AEs leading to permanent discontinuation/withdrawal** | 41 (5) | 55 (7) | 49 (6) | 26 (5) |
| **Fatal SAEs** | 3 (<1) | 5 (<1) | 5 (<1) | 2 (<1) |

#### AEs, adverse events; PBO, placebo; SAEs, serious adverse events; UMEC, umeclidinium; VI, vilanterol
